# On the Use of Arsenic in Teeth

**Published:** 1844-12

**Authors:** Hudson S. Burr


### ARTICLE VIII.

### *On the Use of Arsenic in Teeth.* By Hudson S. Burr, M. D.

I am induced to offer a few physiological and practical remarks on the use of this drug, from seeing an article in our highly appreciated Dental Journal, by Mr. C. L. Norton, of New York, accompanying a red tooth to Professor Harris.

In the first place, we have recourse to arsenic as a dernier resort, knowing it to be effectual when properly applied, and the tooth skilfully attended to after the application. My mode of using it, is after preparing the tooth for its reception, to take a small pledget of cotton, roll it tightly together, take it upon the point of a small plugging instrument, dip it into creosote, and then into arsenic and ascetate of morphia, mixed in one part of the former and two of the latter, place it into the cavity, then covering it over securely with bees-wax and gum mastic melted together, which I keep in small sticks. I say securely, for I have seen extensive sloughing ulcerations from its leaking out of teeth; I never allow it to remain in a tooth longer than twenty-four hours. The tooth should always be finished the next day after its application, except when the bleeding cannot be sufficiently arrested to perform the operation satisfactorily.

What is the physiological action of arsenic? does it kill the nerve in this short time? No, it acts as an escharotic on the exposed ends of the blood vessels and nerve; as a proof that the nerve is not dead, there will still be found some sensibility when the attempt is made to remove the remaining portions of the nerve and blood vessels. After it has been applied, the tooth should be drilled out and the whole root or roots should be plugged up to their extremities, and this can be readily performed upon all the teeth anterior to the molares. When the cavity of a molar tooth is so peculiarly situated as to render it impossible to remove the whole pulp, it must have a hole perforated into the cavity, proper as near the neck as possible, taking care to make it through that part of the tooth that is covered with enamel, as it is far less liable to decay than when the hole is drilled through the ivory of the tooth; this hole may be secured from caries by placing a gold tube in it. At one time I seriously questioned the propriety of ever treating a tooth with arsenic.

I found that teeth, where the nerve was not really exposed, but the tooth so exquisitely sensitive as to exclude the possibility of plugging, that arsenic enabled me to perform the operation the next day with great apparent satisfaction, both to myself and patient; but what was the consequence? The patient would return in a few days, or as late as three months, in one case, with the same tooth aching; in several instances an inability to take any thing hot into the mouth, others any thing cold, some with an intermittent tic douloureux, which I could always trace to the tooth; hence arose the serious question about the propriety of using this powerful drug.

Several teeth having been treated with arsenic, without paying any regard to the condition of their contents, I extracted, sawed them open and carefully examined them; in some I found beautiful specimens of congestion, in some suppuration, others had turned a lively pink color through the whole ivory of the tooth. Now, what occasions this appearance of the teeth? The inflammation having terminated so as to form a sanious secretion or the globuli of the blood being broken up, is taken into the tubeli, thus giving the tooth an injected appearance. When the secretion becomes a dark color, it gives to the tooth a blue appearance in the same manner. The plate of a beautifully magnified tooth may be seen in the new work of Dr. Goddard on teeth.

I have long been looking in our Dental Journal for the experience of some one on the use of arsenic, and have known no better method than to set the example; this, and the fact stated in the commencement, must be the apology for these desultory remarks.

